# Syrosingopine and UK5099 synergistically suppress non-small cell lung cancer by activating the integrated stress response

**DOI:** 10.1038/s41419-024-06821-4

**Published:** 2024-06-19

**Authors:** Yiman Li, Yan Song, Zhijing Shi, Huijie Hou, Yang Yu, Fei Pan, Jinlu Ji, Zhe Chen

**Affiliations:** 1 Department of Respiratory and Critical Care Medicine, Tianjin Medical University General Hospital, Tianjin, China; 2 Department of Geriatrics, Tianjin Geriatrics Institute, Tianjin Medical University General Hospital, Tianjin, China; 3 Health Management Centre, Tianjin Medical University General Hospital, Tianjin, China; 4 Department of Respiratory Medicine, Tianjin Chest Hospital, Tianjin, China

**Keywords:** Non-small-cell lung cancer, Molecular biology

## Abstract

Non-small cell lung cancer (NSCLC) presents a global health challenge due to its low five-year survival rates, underscoring the need for novel therapeutic strategies. Our research explored the synergistic mechanisms of syrosingopine and UK-5099 in treating NSCLC. In vitro experiments showed that the combination of syrosingopine and UK-5099 significantly synergized to suppress NSCLC proliferation. Further experiments revealed that this combination induced cell cycle arrest and promoted apoptosis in NSCLC cells. In vivo experiments demonstrated that the combination of syrosingopine and UK-5099 markedly inhibited tumor growth. Mechanistic studies revealed that this drug combination promoted mitochondrial damage by inducing lactate accumulation and oxidative stress. Additionally, the combination triggered an integrated stress response (ISR) through the activation of heme-regulated inhibitor kinase (HRI). Importantly, our findings suggested that the synergistic suppression of NSCLC by syrosingopine and UK-5099 was dependent on ISR activation. In summary, our study proposed a promising therapeutic approach that involved the combination of Syrosingopine and UK-5099 to activate ISR, significantly hindering NSCLC growth and proliferation.

## Introduction

Non-small cell lung cancer (NSCLC) is one of the most common cancers worldwide with high incidence and mortality rates [1]. Characterized by its heterogeneity and aggressive nature, NSCLC is often diagnosed at an advanced stage, contributing to its notoriously low survival rates [2]. NSCLC presents various challenges due to its resistance to conventional therapies and high recurrence rates [3]. This increasing prevalence and the associated healthcare burden underscore the urgent need for more effective treatment strategies [4].

The integrated stress response (ISR) is a cellular defense mechanism that is activated in response to a variety of stress conditions, such as oxidative stress, endoplasmic reticulum stress, nutrient deprivation, or toxic damage, aiming to restore cellular homeostasis by reducing protein synthesis and enhancing stress remediation pathways. However, ISR can also lead to cell apoptosis with excessive stress [5]. Due to its bidirectional effects on cell survival and cell death, the ISR has gained significant attention as a potential treatment target for several cancers. For instance, ONC201/TIC10 induced cell death in solid tumor cells by triggering an ISR dependent on ATF4 activation [6] and exhibited anti-proliferative effects against a broad range of tumors [7–10]. Researches indicated that compounds such as Indirubin-3’-monoxime [11], GSK2606414 [12], and Trazodone [13] could promote cancer cell death by inhibiting the ISR. These suggest that targeting ISR may be a novel strategy for treating NSCLC.

Syrosingopine and UK5099 have emerged as compounds of interest in cancer research. Syrosingopine, known for targeting monocarboxylate transporters 1 (MCT1) and monocarboxylate transporters 4 (MCT4), has shown potential in disrupting the metabolism of cancer cells. Its role in modulating the Warburg effect, proposes it as a promising treatment in cancer therapy [14]. UK5099, primarily recognized as a mitochondrial pyruvate carrier (MPC) inhibitor [15], alters the metabolic state of cancer cells, thus inducing stress responses [16]. Previous studies have highlighted the potential of these compounds in inducing cytotoxicity in cancer cells, suggesting their utility in targeting cancer cell metabolism and overcoming drug resistance. Their unique mechanisms provide a novel perspective in cancer treatment, particularly in cancers like NSCLC, where metabolism plays a crucial role.

Our study aims to explore the synergistic effects of syrosingopine and UK-5099 in NSCLC, proposing a novel treatment targeting the ISR. We investigated the combined impacts on NSCLC cell including proliferation, cell cycle arrest, apoptosis, and tumor growth. The synergistic effects of the drugs significantly induced oxidative stress and impaired mitochondrial function. Importantly, the combination activated the ISR through HRI, supporting the observed antitumor efficacy. These outcomes suggested that the synergistic impacts of syrosingopine and UK-5099 on NSCLC is mediated through ISR activation, offering a promising therapeutic approach.

## Materials and Methods

### Reagents and Antibody

Chemical compounds utilized in this study included Syrosingopine (Su-3118), UK-5099 (PF-1005023), and ISRIB (HY-12495), all procured from MedChemExpress (Shanghai, NJ, USA). These compounds were prepared following the manufacturer’s guidelines. Specifically, Syrosingopine and UK-5099 were solubilized at a concentration of 50 mM, while ISRIB was dissolved to achieve a 10 mM solution, using Dimethyl Sulfoxide (DMSO) as the solvent. During experimental procedures, these compounds were administered at final concentrations of 5 μM for Syrosingopine, 20 μM for UK-5099, and 10 μM for ISRIB, respectively.

Primary antibodies against Lamin B1 (12987-1-AP) were purchased from ProteinTech (Chicago, IL, USA). Antibodies against Cyclin D1 (E3P5S), p-eIF2α (Ser51) (D9G8), eIF2α (D7D3), β-Actin (8H10D10), and Vinculin (E1E9V) were purchased from Cell Signaling Technology (Danvers, MA, USA). Anti-ATF4 antibody (ab184909) was purchased from Abmart (Shanghai, China).

### Cell lines and cell culture

The human NSCLC cell line H661 was procured from the American Type Culture Collection (ATCC, Manassas, VA, USA), and the human NSCLC cell line PC-9 was generously provided by Dr. Jinling He from the Department of Lung Cancer Surgery at Tianjin Medical University General Hospital, Tianjin, China. All the cell lines were cultured in RPMI 1640 medium (Gibco, USA), enriched with 10% fetal bovine serum (NEWZERUM, S010921, New Zealand) and a 1% penicillin/streptomycin solution (Gibco, 15140-122, USA). The cell lines were then incubated at a consistent temperature of 37 °C in a Thermo incubator, under a humidified atmosphere comprised of 5% CO2.

### Cell viability assay

All NSCLC cells were seeded in 96-well plates and subjected to treatment with varying concentrations of Syrosingopine and UK-5099 for a duration of 48 h. Subsequently, CCK-8 reagent (Vazyme Biotech Co., Ltd, China) was added to facilitate the viability assessment. The presence of viable cells was determined by measuring the absorbance at 450 nm using a specified microplate reader. The data accrued were utilized to calculate the 50% inhibitory concentration (IC_50_) values, employing the software GraphPad Prism 8 (Graph Pad, San Diego, USA).

### Synergy determination with SynergyFinder

H661 and PC-9 cells were seeded into 96-well plates and subsequently treated as elaborated below. Analysis was conducted on either individual inhibitors (Syrosingopine, UK-5099) or their combinations (Syrosingopine and UK-5099), employing the indicated quantities derived from the above cytotoxicity assay. The concentration gradients of Syrosingopine and UK-5099 were pre-established based on each drug’s IC_50_ value, and cell viability was examined at a consistent dilution ratio of the two inhibitors (Syrosingopine concentrations: 0 μM, 1 μM, 5 μM, 10 μM, 20 μM; UK-5099 concentrations: 0 μM, 1 μM, 5 μM, 10 μM, 20 μM, 40 μM). Following a 48 h treatment, the viability of the cells was assessed by measuring absorbance at 450 nm using a microplate reader, as previously detailed. SynergyFinder’s online software (https://synergyfinder.fimm.fi) was utilized to determine drug synergy scores, calculating the “inhibition index” (inhibition index = 100 - Cell Viability) through the response surface model and the zero interaction potency (ZIP) calculation method. ZIP Synergy scores exceeding 0 indicated synergism (highlighted in red regions), and scores surpassing 10 denoted substantial synergistic effects.

### Colony formation assay

To evaluate the colony formation ability following various treatments, 500 cells were precisely seeded into each well of six-well plates, where they were cultured for a period of 2 weeks. After adhering to the wall, the cells were subjected to various treatment for 48 h. The RPMI-1640 culture medium was replaced every three days to maintain optimal growth conditions. Upon completion of the culturing period, a 0.1% crystal violet solution (PH1322, Phygene Scientific, China) was employed to meticulously stain the colonies, allowing for a detailed assessment of colony formation.

### EdU

Cell proliferation assays were conducted using BeyoClick™ EdU Cell Proliferation Kit with Alexa Fluor 488 (C0071S, Beyotime, China). After DMSO, Syrosingopine, UK-5099 or a combination of Syrosingopine with UK-5099 treatment for 48 h, H661 and PC-9 cells were incubated with 10 µM EdU for 2 h at 37 °C. Cells were then fixed with 4% paraformaldehyde in PBS and permeabilized with 0.5% Triton X‐100 at room temperature. Next, cells were incubated in Click Additive Solution, shielded from light, and stained with Hoechst. Stained cells were imaged under a microscope.

### Cell apoptosis assay

Apoptosis in H661 and PC-9 cells was assessed using a kit (BD Pharmingen, 556547). H661 and PC-9 cells were subjected to treatment with DMSO, Syrosingopine, UK-5099, and a combination of Syrosingopine with UK-5099, respectively. In all, 5 × 10^5^ cells were collected and washed twice by cold PBS. Cells were centrifuged at 1500 rpm for 5 min at 4 °C. Then, cells were resuspended in 100 μL of 1× Binding Buffer and incubated with 2.5 μL of Annexin V - FITC and 2.5 μL of PI staining Solution at RT for 15 min. 200 μL of 1× Binding Buffer was added into the mixture. Flow cytometry analysis was performed using the BeckMan CytoFlex (BeckMan, USA) within 1 h.

### Cell cycle

Cell cycle assays were conducted using DNA Content Quantitation Assay (CA1510, Solarbio, China). H661 and PC-9 cells were seeded in six-well plates and treated with DMSO, Syrosingopine, UK-5099, or a combination of Syrosingopine with UK-5099 for 48 h. Cells were fixed in 70% ethanol at 4 °C overnight, then centrifuged, washed with PBS, incubated with 100 μL RNase at 37 °C for 30 min, and stained with 400 μL PI at 4 °C for 30 min in the dark. Measurements were taken using a NovoCyte flow cytometer (Agilent Biosciences, USA), and analysis was performed with NovoExpress software.

### Western blot

Cells were lysed using RIPA buffer containing protease and phosphatase inhibitors (Bimake, USA). Lysates were centrifuged at 12,000 rpm for 15 min, and protein concentration was assessed using a BCA assay kit (CoWin Biosciences, China). The extraction of nucleoplasmic proteins was performed using the cellular nuclear protein and cytoplasmic protein extraction kit (Beyotime, P0027, China). Equal protein amounts were separated by SDS-PAGE and transferred to PVDF membranes. Membranes were blocked with 5% milk and incubated with primary antibodies overnight at 4 °C. Protein bands were visualized using an ECL kit (Millipore, Billerica, MA, USA) and quantified with Image J software.

### Transwell invasion

For the cell invasion study, a Transwell assay was conducted. Initially, cells were placed in 200 μL of serum-free medium, and this mixture was then seeded into an upper chamber that had been pre-coated with Matrigel (Corning, 354234, USA). Simultaneously, 600 μL of 1640 medium containing 10% FBS was added into the lower chamber. Subsequently, various treatments were applied to the cells: they were either cultured with DMSO, Syrosingopine, UK-5099, or a combination of Syrosingopine with UK-5099. After that, the cells on the underside of the membrane were subjected to a fixing and staining process using crystal violet. Finally, these cells were counted and imaged under a microscope to ascertain the results of the invasion study.

### Migration assay

Migration experiments were conducted using scratch assays. Serum-starved H661 and PC-9 cells were seeded into 6-well plates and allowed to grow until they achieved over 80% confluence. A scratch or gap was then carefully introduced at the bottom of each well with a pipette tip to initiate the migration process. Cells were treated with either DMSO, Syrosingopine, UK-5099, or a combination of Syrosingopine and UK-5099. Images of the gap edges were captured systematically at specific time intervals: 0 h, 24 h, and 48 h. For a detailed analysis, these images were processed and evaluated using the ImageJ software (NIH Image J system, Bethesda, MD).

### Mouse xenografts and treatment

Four-week-old female Balb/c nude mice were acquired from Beijing Weitong Lihua (Beijing, China), and housed under specific-pathogen-free (SPF) conditions to ensure a controlled environment. Animal protocols were approved by the Institutional Research Ethics Committee of Tianjin Medical University (approval number: TMUaMEC 2023038). For the initiation of tumor formation, 5 × 10^6^ PC-9 cells were subcutaneously inoculated into the Balb/c nude mice. Once the tumor volumes reached 50 mm^3^, the mice were methodically divided into four groups, each consisting of six mice: Control (PBS), Syrosingopine (7.5 mg/kg), UK-5099 (3 mg/kg), and a combination of Syrosingopine and UK-5099. Treatments, including Syrosingopine, UK-5099, or PBS, were administered to the mice through intraperitoneal injections continuously for 14 days. Throughout this period, observations were diligently made, recording the tumor volume and weight for subsequent analysis.

### Histopathological analysis

Mice were sacrificed and various organs including brain, heart, lungs, liver, spleen and kidneys were harvested. The organs were fixed in poly formaldehyde and embedded in paraffin, and cut into 3um thick sections. The sections were stained with hematoxylin-eosin (H&E) and examined for histopathological changes under the microscope.

### Mice serum biochemical analysis

Blood was collected through retro-orbital bleeding in the different treatment groups of nude mice. Serum samples were collected by centrifugation at 4,000 rpm for 15 min at 4 °C and was recovered for biochemical analysis. The Serum levels of aspartate aminotransferase (AST) (Rayto, S03040, China) and alanine aminotransferase (ALT) (Rayto, S03030, China) were measured to evaluate liver function. Renal function was investigated by quantifying blood urea nitrogen (BUN) (Rayto, S03036, China) and creatinine (CR) (Rayto, S03076, China) levels.

### Reactive oxygen species

The intracellular reactive oxygen species levels were meticulously analyzed using a Reactive Oxygen Species Assay Kit (Beyotime, S0033, China), adhering strictly to the manufacturer’s instructions. H661 and PC-9 cells were treated with DMSO, Syrosingopine, UK-5099, or a combination of Syrosingopine and UK-5099. These treated cells were then isolated and suspended in diluted DCFH-DA. Subsequently, the cell suspensions were incubated at 37 °C for 20 min in an environment with 5% CO2. Following incubation, the cells were washed with serum-free medium to remove any residues. Finally, the cellular fluorescence, indicative of ROS levels, was immediately assessed through flow cytometry analysis.

### Lipid peroxidation assay

The Lipid Peroxidation MDA Assay Kit (Beyotime, S0131S, China) was utilized to assess the production of malondialdehyde (MDA) in various groups of H661 and PC-9 cells, which included treatments with DMSO, Syrosingopine, UK-5099, or a combination of Syrosingopine and UK-5099. Proteins were extracted from the collected cells and the concentration was accurately measured. Following this, 100 µL of the samples were carefully mixed with 200 µL of MDA detection fluid and then incubated at a temperature of 100 °C for a duration of 15 min. This was followed by centrifugation at a force of 1,000 g for 10 min. In the concluding step, 200 µL of the supernatant was transferred to a 96-well plate, and absorbance was measured precisely at 532 nm. Based on these measurements, the level of lipid peroxidation was then meticulously calculated, being expressed in terms of nmol/mg protein.

### Superoxide dismutase

The Total Superoxide Dismutase Assay Kit from Beyotime (S0101S, China) was utilized to evaluate the Superoxide Dismutase activity in cells. Briefly, H661 and PC-9 cells were treated with DMSO, Syrosingopine, UK-5099, or a combination of Syrosingopine and UK-5099 for 48 h. Proteins were extracted from the collected cells and the concentration was accurately measured. Subsequently, 20 µL of the cell samples were added to a 96-well plate along with 180 µL of superoxide dismutase (SOD) detection fluid and incubated at 37 °C for 30 min, and absorbance was measured precisely at 450 nm. Based on these measurements, the level of lipid peroxidation was calculated and expressed as nmol/mg protein.

### Mitochondrial membrane potential

The JC-1 Mitochondrial Membrane Potential Assay Kit (MCE, HY-K0601, USA) was used to measure mitochondrial membrane potential. H661 and PC-9 cells were treated with DMSO, Syrosingopine, UK-5099, or a combination of Syrosingopineand UK-5099 for 48 h. After the treatment, cells were washed with PBS and stained with JC-1 before incubating at 37 °C for 30 min. Then cells were collected, washed twice with PBS and resuspended for flow cytometry analysis.

### ATP

The ATP level of H661 and PC-9 was measured with an ATP kit (Beyotime, S0026, Haimen, China). Briefly, H661 and PC-9 cells were treated with DMSO, Syrosingopine, UK-5099, or a combination of Syrosingopine and UK-5099 for 48 h. Cell samples (100 µL) were mixed with ATP detection fluid (100 µL) and incubated at room temperature for 3 min. The lysate were centrifuged at 12,000 × *g* for 5 min at 4 °C. The ATP content was read by a microplate reader with luminometer function.

### Glycolysis assay

The Glycolysis Assay Kit (Abcam, ab197244, USA) was used to measure extracellular acidification due to lactate production during glycolysis. H661 and PC-9 cells were cultured in 96-well plates and treated with DMSO, Syrosingopine, UK-5099, or a combination of Syrosingopine and UK-5099 for 48 h. Subsequently, to purge CO2, the cells were placed in a CO2-free incubator maintained at 37 °C for a period of 3 h. Following this, 150 μl of Respiration Buffer and 10 μl of the Glycolysis Assay Reagent were added. The plates were then transferred to a TECAN fluorescent spectroscope. Here, fluorescence measurements (Ex 380 ± 40 nm Em 620 ± 10 nm) were diligently taken at intervals of 1.5 min over a span of 1.5–2 h.

### O_2_ consumption assay

The Extracellular Oxygen Consumption Assay Kit (Abcam, ab197242, USA) was used for real-time kinetic analysis of oxygen consumption. H661 and PC-9 cells were cultured in 96-well plates and treated with DMSO, Syrosingopine, UK-5099, or a combination of Syrosingopine and UK-5099 for 48 h. Afterwards, the medium was replaced with a serum-free culture medium, and 10 μl of the Extracellular Oxygen Consumption Reagent was added. Prior to oxygen determination, 100 μl of High Sensitivity Mineral Oil was added on top of the assay medium to limit oxygen diffusion into the medium. O2 consumption was measured using a TECAN fluorescent spectroscope, with readings taken (Ex 380 ± 20 nm, Em 650 ± 20 nm) every 1.5 min for a duration of 1.5–2 h.

### RNA extraction, RT-qPCR, and RNA-seq

Total RNA from H661 and PC-9 cells was extracted using TRIzol reagent (Invitrogen, USA) and reverse-transcribed into cDNA with a cDNA synthesis kit (Vazyme Biotech, China). PCR was performed with primers and SYBR Green (Vazyme Biotech, China). PCR conditions: 95 °C for 30 s, followed by 40 cycles of 95 °C for 3 s and 60 °C for 10 s. Primers are listed in Supplementary Table 1. The comparative Ct method was applied for mRNA quantification, normalizing each gene to GAPDH and expressing fold changes relative to the control group. For RNA-seq, RNA samples were sequenced by Lianchuan (Hangzhou, China). The genes with the parameter of false discovery rate below 0.05 and absolute fold change ≥2 were considered differentially expressed genes. Differentially expressed genes were subjected to enrichment analysis of KEGG functions and GO pathways using the OmicStudio tools at https://www.omicstudio.cn/tool.

### Transient transfection and lentivirus infection

siRNAs were synthesized by Integrated Biotech Solutions (Shang Hai, China), and the sequences are listed in Supplementary Table 2. A pool of the three separate siRNAs, which were designed to target a single gene, were delivered into H661 or PC-9 cells using lipofectamine 2000, and the final concentration of siRNAs was 100 nM. Alongside, a scramble siRNA was also employed, at a consistent concentration of 100 nM, serving as a negative control (si-NC) to validate the specificity and efficiency of the gene-silencing process. The lentivirus eIF2α^WT^ and eIF2α^S51A^ were generously provided by Dr. Jinling He. H661 and PC-9 cells were infected with the indicated lentiviruses, and stable cell populations were established using puromycin.

### Statistics and data analysis

Data compilation and statistical computations were executed using GraphPad Prism 8 software. All experiments were conducted in triplicate, with outcomes represented as mean ± SD. Intergroup differences were analyzed through the T-test. A *p*-value below 0.05 was deemed statistically significant.

## Result

### Synergistic suppression of NSCLC proliferation by Syrosingopine and UK-5099 in vitro

Given that Syrosingopine targets MCT1 and MCT4, and UK-5099 targets MPC1 and MPC2, we conducted RT-qPCR to assess the expression of these targets in NSCLC cell lines, including H1792, A549, H23, H661, H1299, H1975, PC-9, H2030, and YTMLC-90. H661 showed the highest expression of MCT1, MCT4, MPC1, and MPC2, while PC-9 ranked second in terms of MCT4, MPC1, and MPC2 expression, and also exhibited notable expression levels of MCT1 (Fig. S1). Therefore, based on the results, H661 and PC-9 were selected for further experimentation.

**Fig. 1:**
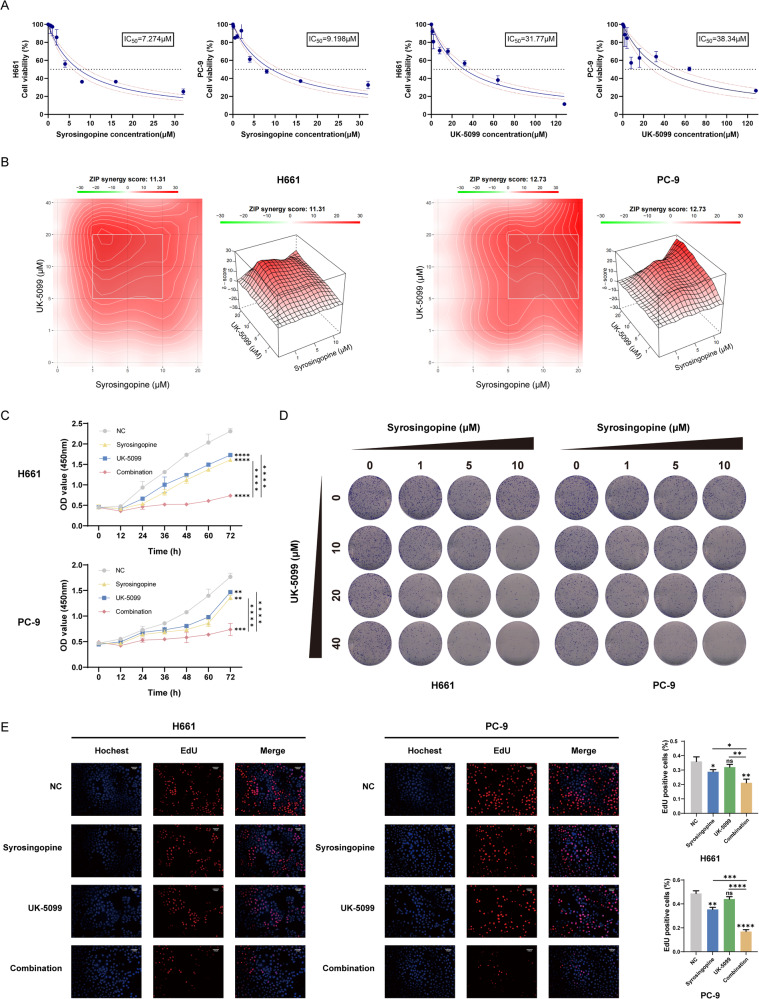
The combinations of Syrosingopine and UK-5099 exhibited a synergistic suppression of NSCLC proliferation in vitro. **A** Dose-response curves for Syrosingopine and UK-5099 in H661 and PC-9. IC_50_ values were determined using the CCK-8 assay after 48 h of treatment (*n* = 3). **B** Heatmaps of drug combination responses. ZIP Synergy scores were calculated with Synergyfinder software. Scores > 0 indicated synergism and scores > 10 were considered strongly synergistic (*n* = 6). **C** Cell proliferation assays of H661 and PC-9 cell lines treated with Syrosingopine, UK-5099, and their combination. Cell viability was measured using an CCK-8 assay at various time points (0, 12, 24, 36, 48, 60, and 72 h) (*n* = 3). **D** Colony formation assays showed the effects of the various concentrations of Syrosingopine and UK-5099 on the clonogenic survival of H661 and PC-9 cells (*n* = 3). **E** EdU incorporation assays were used to evaluate cell proliferation in H661 and PC-9 cells treated with Syrosingopine, UK-5099, or their combination (*n* = 3). (*P*-values: not significant (ns) *p* > 0.05, *p ≤ 0.05, **p ≤ 0.0,1 ***p ≤ 0.001, ****p ≤ 0.0001).

The in vitro experiments demonstrated a synergistic effect of Syrosingopine and UK-5099 in inhibiting the proliferation of NSCLC cells. Initially, the sub-cytotoxic concentrations of Syrosingopine and UK-5099 were determined for H661 and PC-9 cell lines using a CCK-8 assay. The observed anti-proliferative effects were concentration-dependent, as evidenced by the IC_50_ values in Fig. 1A. Further investigation into the synergistic interaction of these drugs was conducted through a refined concentration gradient analysis, employing the ZIP drug synergy model in SynergyFinder software. This analysis revealed average and maximum drug interaction contributions of 11.31% and 16.97%, respectively, in H661 cells and 12.73% and 19.04% in PC-9 cells, as shown in Fig. 1B. Notably, these ZIP synergy scores, exceeding the 10% threshold, highlight the potent synergistic impact of the drug combination. The effectiveness of Syrosingopine (5 μM) and UK-5099 (20 μM) in reducing cell viability was further substantiated by CCK-8 assay results (Fig. 1C), indicating a significant decrease in cell viability over time. Colony formation assays demonstrated a marked reduction in clonal growth in both cell lines (Fig. 1D), corroborating the combination suppressed the colony formation ability of H661 and PC-9 cells. Additionally, EdU staining assays provided visual confirmation of reduced cell proliferation (Fig. 1E). These in vitro results consistently indicate that Syrosingopine and UK-5099 significantly hinder the proliferation of NSCLC cells, offering promising insights into potential therapeutic strategies.

### Impact of Syrosingopine and UK-5099 combination on cell cycle arrest and apoptosis in NSCLC cells

**Fig. 2:**
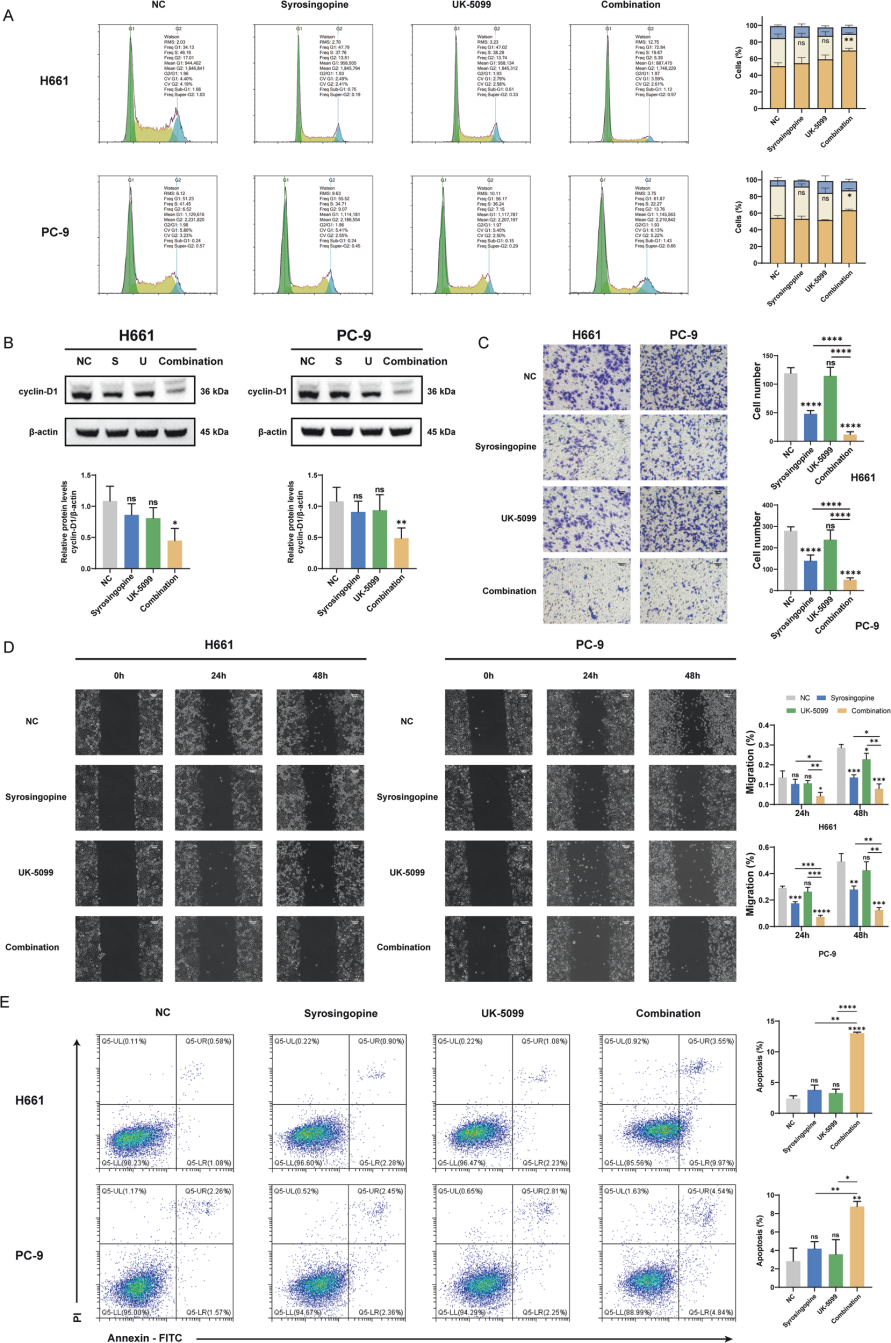
The combinations of Syrosingopine and UK-5099 induced cell cycle arrest and apoptosis. **A** Cell cycle analysis showing the distribution of H661 and PC-9 cells in different cell cycle phases post-treatment with Syrosingopine, UK-5099, or their combination (*n* = 3). **B** Western blot detection of the level of cyclin D1 protein after treatment with Syrosingopine, UK-5099, or their combination (*n* = 3). **C** The effect of Syrosingopine and UK-5099 on the invasion capabilities of H661 and PC-9 cells was determined by Transwell invasion assays (*n* = 3). **D** The effect of Syrosingopine and UK-5099 on the migratory capabilities of H661 and PC-9 cells was determined by a scratch wound assay (*n* = 3). **E** Flow cytometric analysis of apoptosis via Annexin V-FITC/PI staining in H661 and PC-9 cells following exposure to Syrosingopine, UK-5099, or their combination (*n* = 3). (*P*-values: not significant (ns) *p* > 0.05, *p ≤ 0.05, **p ≤ 0.01, ***p ≤ 0.001, ****p ≤ 0.0001).

Given the role of the combination in regulating cell proliferation, we investigated whether the combination of Syrosingopine and UK-5099 affects the cell cycle. Cell cycle assays indicated that the treatment induced G0/G1 phase arrest in both H661 and PC-9, as depicted in Fig. 2A. Further, Western blot analysis showed a noticeable downregulation of Cyclin D1 protein expression in these cells (Fig. 2B), aligning with the observed cell cycle arrest. In addition, in vitro wound healing and Transwell assays demonstrated that the combination of Syrosingopine and UK-5099 reduced the invasive (Fig. 2C) and migratory (Fig. 2D) potential of NSCLC cells. Furthermore, the extent of apoptosis induced by the treatments was quantified through flow cytometric analysis. The findings revealed that the combined treatment significantly enhanced apoptosis in both H661 and PC-9 cells compared to individual treatments (Fig. 2E). In summary, the combination of Syrosingopine and UK-5099 was found to exert a robust synergistic inhibitory effect on NSCLC cells in vitro. This effect manifested as induced apoptosis, G0/G1 phase arrest, and a marked reduction in the migration and invasion abilities of the cells.

### In vivo inhibition of tumor growth by the combination of Syrosingopine and UK-5099

**Fig. 3:**
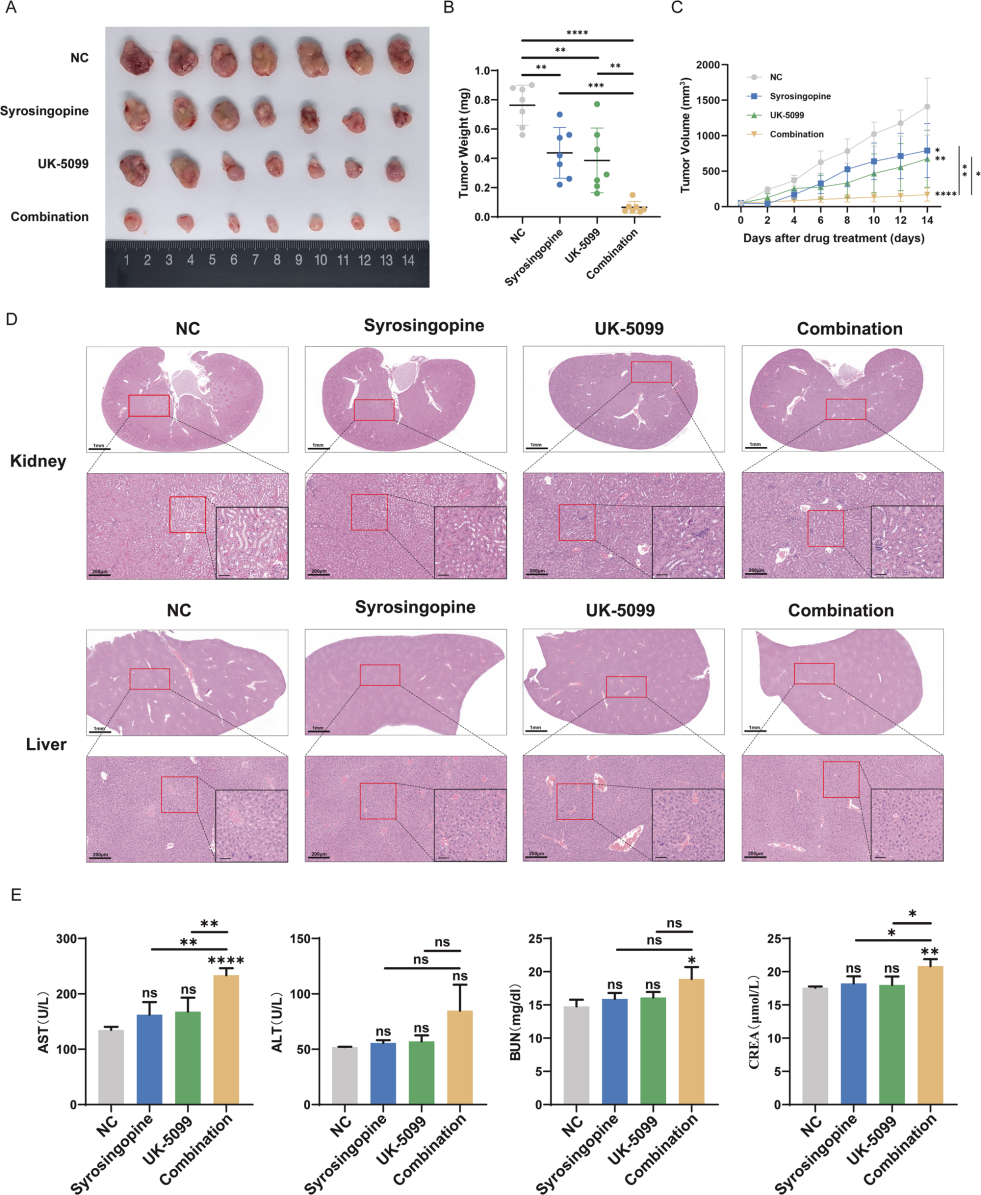
The combinations of Syrosingopine and UK-5099 inhibited tumor growth in vivo. **A** The representative images of tumors excised from mice treated with NC, Syrosingopine, UK-5099, or the combination of Syrosingopine and UK-5099 are displayed. The tumors were collected and photographed at the end of the treatment period (*n* = 7). **B** The tumor weights were immediately measured after excision (*n* = 7). **C** The tumor volume was monitored over the treatment period and plotted to show the growth kinetics of each treatment group (*n* = 7). **D** H&E staining of liver and kidney tissues from mice treated with NC, Syrosingopine, UK-5099, or the combination of these drugs was performed. The representative images illustrate the tissue architecture and cellular morphology for each treatment group (*n* = 5). **E** The test result of liver and kidney functions for each treatment group (*n* = 7). (*P*-values: not significant (ns) *p* > 0.05, *p ≤ 0.05, **p ≤ 0.01, ***p ≤ 0.001, ****p ≤ 0.0001).

To evaluate the synergistic effects of Syrosingopine and UK-5099 in vivo, a subcutaneous tumor model was established in nude mice using PC-9 cells. After tumor volumes reached 50 mm^3^, the mice were intraperitoneally injected with either PBS, Syrosingopine (7.5 mg/kg) alone, UK-5099 (3 mg/kg) alone, or the combination of both drugs at the same doses. Tumor growth was closely monitored throughout the treatment period. At the end of the experiment, mice were euthanized, and tumors were excised, with representative images shown in Fig. 3A. The group receiving the combination treatment displayed a significant inhibition of tumor growth. A notable reduction in tumor weight evidenced this compared to the control and the single-drug treatment groups (Fig. 3B). In contrast, the control group and the groups treated with either Syrosingopine or UK-5099 alone showed progressive tumor enlargement (Fig. 3C). Tumors from the combination treatment group were substantially smaller than those in the single-drug treatment groups.

Additionally, histological analysis was conducted on liver, kidney, brain, heart, and spleen specimens from the four groups. H&E staining was utilized to assess the potential toxic side effects of the treatments. No significant pathological changes were observed in the liver, kidney (Fig. 3D), brain, heart, and spleen (Fig. S2) in any of the groups compared to the control. Furthermore, we collected serum from each group of mice post-treatment for liver and kidney function tests. Compared to the control group, both the monotherapy groups and the combination therapy group showed varying degrees of elevation in AST, ALT, BUN, and CR levels, with the increases in the combination group being particularly notable, where AST and ALT levels approached the upper limits of the normal range. However, the levels of AST, ALT, BUN, and CR were all within normal ranges in the four groups (Fig. 3E and Table S3). Despite the elevated liver and kidney function markers, the H&E staining results indicate that the combined treatment of Syrosingopine and UK-5099 did not cause significant toxic side effects to the nude mice under our experimental conditions.

### Mitochondrial damage and metabolic dysfunction induced by Syrosingopine and UK-5099 through lactate accumulation and oxidative stress

**Fig. 4:**
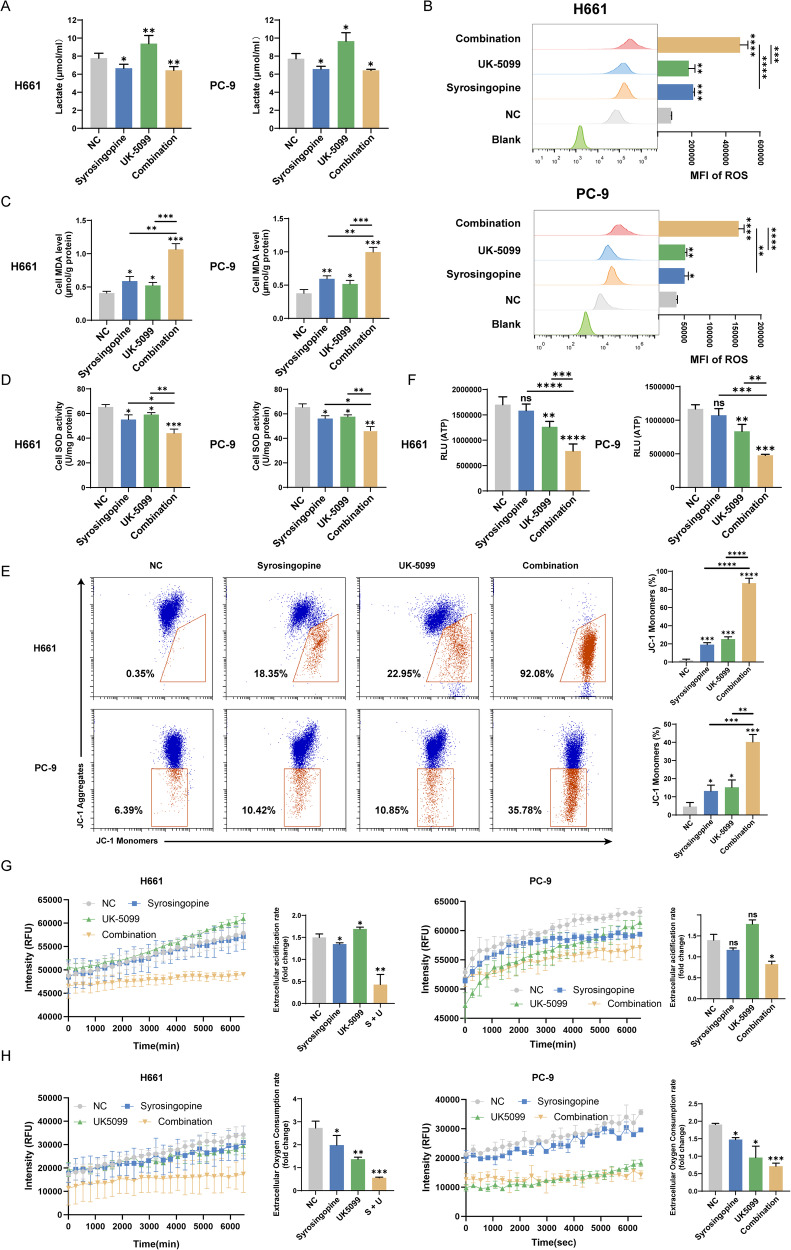
The combinations of Syrosingopine and UK-5099 promoted mitochondrial damage and metabolic dysfunction by inducing lactate accumulation and oxidative stress. **A** Lactate secretion in the cell medium was measured after various treatments (*n* = 3). **B**–**D** The effects of syrosingopine, UK-5099, or their combination on ROS (**B**), MDA (**C**), and SOD (**D**) levels were assessed in H661 and PC-9 cells (*n* = 3). **E** Mitochondrial membrane potential was detected by JC-1 staining following various treatments (*n* = 3). **F** Intracellular ATP levels were detected using an ATP assay kit in H661 and PC-9 cells (*n* = 3). **G** Glycolysis in H661 and PC-9 cells treated with syrosingopine, UK-5099, or their combination was measured by assessing cytoplasmic acidification (*n* = 3). **H** Oxygen consumption in H661 and PC-9 cells treated with syrosingopine, UK-5099, or their combination was detected by oxygen consumption analysis (*n* = 3). (*P*-values: not significant (ns) *p* > 0.05, *p ≤ 0.05, **p ≤ 0.01, ***p ≤ 0.001, ****p ≤ 0.0001).

We first measured extracellular lactate levels. Notably, the combination treatment group and the group treated with Syrosingopine alone showed a significant reduction in lactate levels in the culture medium, whereas an increase in lactate was observed in the UK-5099-treated group (Fig. 4A). This suggests enhanced lactate accumulation within the cells, a condition known to induce oxidative stress [17, 18]. To further assess the oxidative stress induced by these treatments, we evaluated intracellular levels of ROS, SOD, and the oxidative stress marker MDA in H661 and PC-9 cells. The results demonstrated a considerable elevation in ROS (Fig. 4B), and MDA accumulation (Fig. 4C), along with a reduction in SOD (Fig. 4D) activity in the cells treated with the combination of Syrosingopine and UK-5099, as compared to the control group and the groups treated with each drug separately.

Given the link between oxidative stress and mitochondrial dysfunction, we examined mitochondrial membrane potential (MitoMP) using JC-1 fluorescent dye. Treatment with the drug combination led to a decrease in red fluorescence intensity (JC-1 aggregates) and an increase in green fluorescence (JC-1 monomers) (Fig. 4E), indicating a disruption in mitochondrial membrane potential. Additionally, ATP levels were significantly reduced in cells treated with the combination of both drugs (Fig. 4F), suggesting an impact on cellular energy production. Further investigations into extracellular acidification rate (ECAR) and oxygen consumption rate (OCR) revealed that the combined treatment of Syrosingopine and UK-5099 diminished both (Fig. 4G, H). These findings indicate a substantial inhibition of glycolysis and mitochondrial respiration, leading to altered energy metabolism in NSCLC cells. In summary, the combination of Syrosingopine and UK-5099 induces significant oxidative stress and disrupts energy metabolism in NSCLC cells, promoting mitochondrial damage.

### Activation of the integrated stress response by Syrosingopine and UK-5099 via HRI in NSCLC cells

**Fig. 5:**
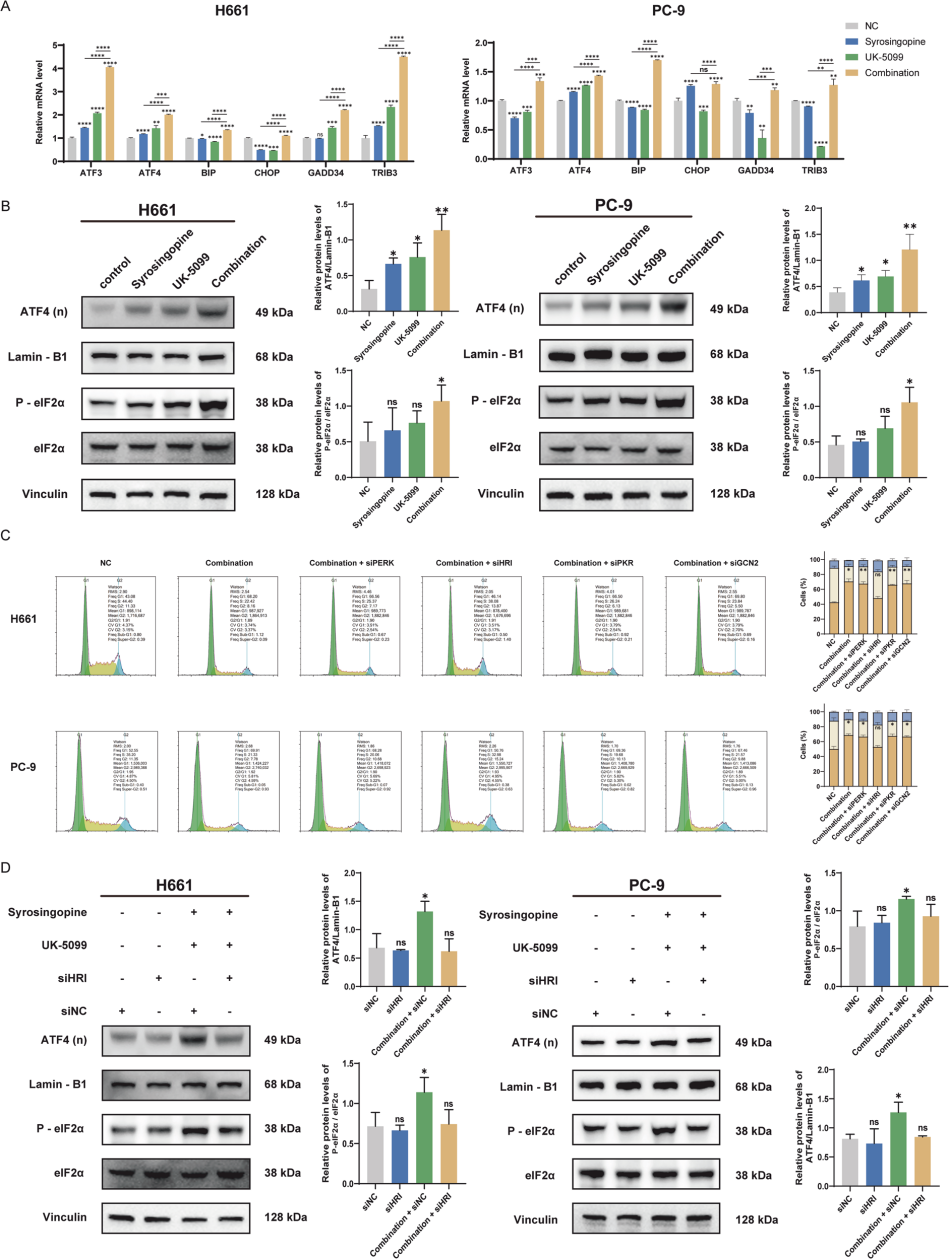
The combination of Syrosingopine and UK-5099 triggers an integrated stress response through HRI activation. **A** RT-qPCR to confirm the expression of genes involved in ISR (*n* = 3). **B** The level of ATF4, P-eIF2α, and eIF2α in H661 and PC-9 after various treatments was analyzed by western blot (*n* = 3). **C** Analysis of cell cycle distribution in H661 and PC-9 cells treated with Syrosingopine and UK-5099 after knockdown of four eIF2α kinases (*n* = 3). **D** Western blot analysis of ATF4, P-eIF2α, and eIF2α levels in H661 and PC-9 cells treated with Syrosingopine and UK-5099 following knockdown of four eIF2α kinases (*n* = 3). (*P*-values: not significant (ns) *p* > 0.05, *p ≤ 0.05, **p ≤ 0.01, ***p ≤ 0.001, ****p ≤ 0.0001).

Next, our study investigated the activation of the ISR pathway in NSCLC cells treated with the combination of Syrosingopine and UK-5099. The ISR pathway responds to various stresses, including oxidative stress, and is crucial for cellular adaptation and survival. We first assessed the mRNA expression levels of key ISR downstream effectors in H661 and PC-9 cells. The results revealed a significant increase in the mRNA levels of ATF3, ATF4, BIP, CHOP, TRIB3, and GADD34 following combination therapy, compared to cells treated with DMSO or either drug alone (Fig. 5A), which suggests the activation of the ISR pathway. To further confirm ISR activation, we measured phosphorylated eIF2α (Ser51) levels and ATF4 nuclear accumulation, which are established indicators of ISR engagement. In cells treated with the combination therapy, we observed notable increases in ATF4 nuclear accumulation and phosphorylated eIF2α levels (Fig. 5B), indicating effective ISR activation. Next, we performed RNA-Seq on the H661 combination group and the H661 NC group. The RNA-seq results identified 973 upregulated and 1,655 downregulated genes in cells treated with the combination compared to the control group (Fig. S3A). Next, we performed KEGG (Fig. S3B) and GO (Fig. S3C) enrichment analysis of these differentially expressed genes. The KEGG and GO enrichment of pathways such as the cell cycle, p53 signaling pathway, apoptosis, transcriptional misregulation in cancer, and metabolic pathways, along with GO categories like protein binding, cell cycle, and ATP binding, indirectly supports the activation of ISR-related processes.

Given that ISR can be mediated by four kinases—PERK, HRI, PKR, and anS5d GCN2—we next sought to identify the specific kinase responsible for ISR activation in our study. Through selective knockdown experiments of each kinase (Fig. S4), we determined that the absence of HRI, unlike the other kinases, prevented cell cycle arrest following combination therapy (Fig. 5C). HRI-deficient cells did not exhibit increased levels of ATF4 nuclear accumulation or phosphorylated eIF2α upon treatment with the drug combination (Fig. 5D). These findings collectively indicate that the combination of Syrosingopine and UK-5099 triggers ISR activation predominantly through HRI in NSCLC cells.

### The synergistic suppression of Syrosingopine and UK-5099 in NSCLC depends on the integrated stress response

**Fig. 6:**
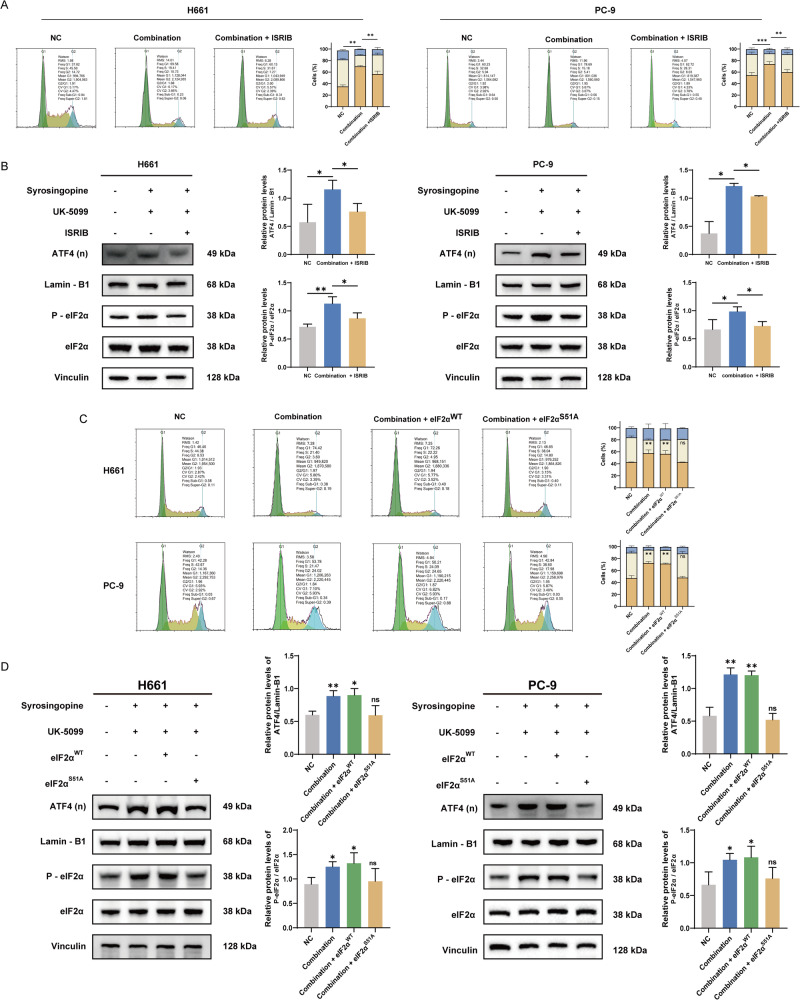
The synergistic suppression effect of the combination of Syrosingopine and UK-5099 is dependent on the ISR. **A** Cell cycle analysis assessed the impact of ISRIB intervention on H661 and PC-9 cells (*n* = 3). **B** Western blot analysis was performed to determine the levels of ATF4, P-eIF2α, and eIF2α in H661 and PC-9 cells after ISRIB intervention and treatment with Syrosingopine and UK-5099 (*n* = 3). **C** Cell cycle analysis revealed that the mutation of Ser51 to Ala51 in eIF2α significantly inhibited the response to Syrosingopine and UK-5099 in H661 and PC-9 cells, compared to cells with wild-type eIF2α (*n* = 3). **D** Western blot analysis showed that he mutation of Ser51 to Ala51 in eIF2α significantly reduced ATF4 and P-eIF2α expression following treatment with Syrosingopine and UK-5099 in H661 and PC-9 cells (*n* = 3). (*P*-values: not significant (ns) *p* > 0.05, *p ≤ 0.05, **p ≤ 0.01, ***p ≤ 0.001, ****p ≤ 0.0001).

Our study aimed to ascertain if the synergistic suppression effect observed with the combination of Syrosingopine and UK-5099 in NSCLC cells is contingent upon the activation of the ISR. To this end, rescue experiments were performed using ISRIB, a pharmacological inhibitor of ISR. Treatment with ISRIB effectively reversed the G0/G1 arrest induced by the drug combination (Fig. 6A), indicating a direct correlation between ISR activity and cell cycle arrest. Furthermore, Western Blot analysis revealed that the phosphorylated eIF2α (Ser51) level and the nuclear accumulation of ATF4, both key markers of ISR activation, were reduced upon ISRIB treatment (Fig. 6B).

Since the phosphorylation of Ser51 in eIF2α is a critical event for ISR activation, we introduced wild-type eIF2α (eIF2α^WT^) or the mutant eIF2α^S51A^ into H611 and PC-9 cells to assess the potential effect of the mutated eIF2α on cell viability. The eIF2α^S51A^ mutation, which prevents phosphorylation at Ser51, led to a marked deactivation of ISR and an increase in the S phase of cells treated with the combination of Syrosingopine and UK-5099 (Fig. 6C). Correspondingly, phosphorylated eIF2α (Ser51) levels and ATF4 nuclear accumulation were decreased (Fig. 6D). These results collectively demonstrate that the synergistic suppression effect of Syrosingopine and UK-5099 on NSCLC cells depends on the ISR pathway. These data suggest that the activation of ISR is a primary mechanism driving the antitumor effects of the combination of syrosingopine and UK-5099.

## Discussion

**Fig. 7:**
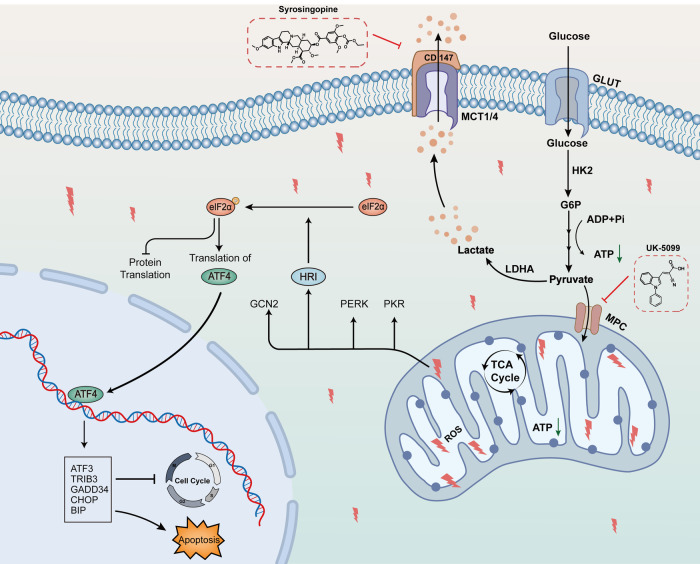
Mechanism diagram. Syrosingopine selectively inhibits MCT1/MCT4, reducing lactate efflux from cells. Combined with UK-5099, an inhibitor of mitochondrial pyruvate transport, this leads to an intracellular accumulation of lactate, causing mitochondrial dysfunction and the generation of excessive ROS. This dual inhibition disrupts both aerobic glycolysis and oxidative phosphorylation within tumor cells. The resultant oxidative stress activates the ISR, inhibiting proliferation, migration, and invasion while inducing apoptosis and cell cycle arrest.

To our knowledge, this is the first study to explore the therapeutic efficacy of the combination of syrosingopine and UK-5099 in NSCLC. In our study, syrosingopine combined with UK-5099 synergistically inhibited the proliferation, migration, and invasion of NSCLC cells, promoted apoptosis, and caused cell cycle arrest at the G0/G1 phase. In animal experiments, the combination of syrosingopine and UK-5099 significantly inhibited the growth of subcutaneous tumors in vivo, without observing significant toxic side effects. The combination of syrosingopine and UK-5099 significantly increased ROS and MDA levels and decreased SOD activity, indicating the induction of oxidative stress. The combination also led to a reduction in mitochondrial membrane potential and ATP levels, and simultaneously inhibited the extracellular acidification rate and oxygen consumption rate. Further exploration of the mechanism revealed that the combination significantly promoted the expression of downstream genes related to the ISR, increased the levels of cytoplasmic p-eIF2α and the accumulation of ATF4 in the nucleus, and validated through rescue experiments that this synergistic antitumor effect is dependent on the ISR activated by HRI (Fig. 7).

Syrosingopine, used as an anti-hypertensive drug for over 40 years [19], has recently revealed its potential in cancer treatment. Don Benjamin et al. first reported the synthetic lethality effect of syrosingopine combined with metformin in granulocytic leukemia, sarcoma, and multiple myeloma, providing crucial clues for subsequent antitumor studies [20]. Then, the same team delved deeper into the mechanism of syrosingopine, discovering its dual inhibition of MCT1 and MCT4. This dual inhibition blocks the extrusion of lactate and H + , leading to intracellular lactate accumulation, effectively inhibiting tumor growth [21]. Further studies also revealed that syrosingopine regulates metabolic pathways in breast cancer and pharyngeal squamous cell cancer, primarily by reducing lactate efflux and enhancing oxidative phosphorylation, thereby inducing apoptosis and inhibiting tumor cell proliferation [22]. Similar effects on tumor suppression through metabolic regulation were observed in thyroid cancer [23]. Moreover, syrosingopine could synergize with chemotherapy drugs [14] or endocrine therapy drugs [24], enhancing the killing effect on tumor cells. These studies not only demonstrated the potential effects in tumor therapy but also explored its synergistic effect with other treatments. Similar results were also observed in our study, noting that syrosingopine inhibited proliferation, migration, invasion, and tumor growth in non-small cell lung cancer cells to a certain extent.

UK-5099, an inhibitor targeting the mitochondrial pyruvate carrier [25], disrupts the process of pyruvate entering the mitochondria, affecting cellular energy metabolism [15]. In prostate cancer, UK-5099 effectively induced cell cycle arrest at the G0/G1 phase and inhibited cell proliferation by weakening mitochondrial oxidative phosphorylation and promoting a metabolic shift towards glycolysis. However, it was found that this metabolic reprogramming led to increased chemotherapy resistance [26]. Moreover, in breast cancer, inhibiting the mitochondrial pyruvate carrier suppressed tumor cell proliferation and enhanced sensitivity to radiotherapy both in vitro and in vivo [27]. Furthermore, Hongbo Zou et al. found that overexpression of MPC1 could promote proliferation, invasion, migration, and subcutaneous tumor growth in NSCLC [28]. In our study, we found that UK-5099 inhibited tumor growth to a certain extent in vivo, however, it showed no significant influence on NSCLC cell proliferation, apoptosis, migration, and invasion in vitro. Research on UK-5099 as an antitumor drug is still in its early stages, and its specific mechanisms of action, optimal treatment regimens, and potential side effects require further exploration and verification through more studies.

Furthermore, our study focused on the metabolism of the syrosingopine and UK-5099 combination. Research showed that excessive lactate accumulation [29, 30] and oxidative stress [31, 32] led to mitochondrial damage. The dysfunction of mitochondria disrupted normal metabolic processes, which contributed to the inhibition of tumor cell proliferation [33]. Many studies have substantiated the suppressive impact of oxidative stress and metabolic disturbances on tumor proliferation and progression. Jiaqi Li et al. reported that the Formosanin C hindered MCT4/CD147, leading to the accumulation of intracellular lactate, which provoked oxidative stress, resulting in inhibition of tumor proliferation [34]. Zhu Z et al. proposed that Anlotinib combined with metformin enhanced oxidative stress in NSCLC cells, and inhibited the mTOR pathway, demonstrating a synergistic antitumor effect [35]. The combination of syrosingopine and UK-5099 in our study exacerbated oxidative stress, led to a significant increase in ROS and MDA levels, with a decrease in SOD activity. Furthermore, our results revealed a marked reduction in mitochondrial membrane potential and ATP levels, significantly affecting cellular respiration by reducing the extracellular acidification rate and oxygen consumption rate. These effects collectively contributed to inhibiting the progression of NSCLC.

The ISR facilitates cell survival and homeostasis by modulating mRNA translation to inhibit global protein synthesis while selectively increasing the production of certain proteins, such as ATF4, that can promote cellular adaptation to stress [36, 37]. In lung adenocarcinoma patients, increased ISR activation correlated with regions of higher proliferation, invasiveness, and tumor growth [38]. Additionally, KRAS promoted asparagine biosynthesis in response to nutrient stress by regulating ATF4 transcription [38]. However, excessive activation of ISR can also lead to tumor cell apoptosis and inhibition of tumor cell proliferation, which present a compelling target for cancer therapy [39, 40]. In our study, we observed that the combination notably enhanced the expression of ISR-associated genes, increased the levels of cytoplasmic p-eIF2α and the accumulation of ATF4 in the nucleus. The results suggested that the ISR activation induced by the combination of syrosingopine and UK-5099 might inhibit the progression of NSCLC. Numerous studies have demonstrated that activating the ISR can inhibit tumor progression. Jirapat Namkaew et al. reported that mifepristone could enhance the efficacy of cisplatin-based therapy for NSCLC by triggering the ISR [41]. Additionally, activators of eIF2α kinase like CCT020312 [42], BTdCPU, and histidine [43] enhanced ISR signaling which led to the depletion of cyclin D dependent on eIF2α and resulted in tumor cell cycle arrest at the G1/S phase. Tsaytler P et al. found that guanabenz and its derivative Sephin1 exerted antitumor effects by inhibiting the stress-induced phosphatase GADD34 [44]. Gang Ma et al. reported that in gastric cancer cells, CDO1-induced oxidative stress excessively activated ISR, leading to the inhibition of gastric cancer cell proliferation [45]. Denis M et al. indicated that in multiple myeloma, inhibiting eIF2α phosphorylation aided tumor cell survival, while sustained ISR activation could enhance the efficacy of proteasome inhibitor treatment [46]. In our study, we validated that the combination of syrosingopine and UK-5099 activated a sustained activation of the ISR through HRI kinase, leading to the apoptosis of NSCLC cells and inhibition of their proliferation. Moreover, rescue experiments further elucidated that the synergistic inhibition of tumor cell proliferation by the drug combination depended on activating ISR.

The limitations of our study should be highlighted. Firstly, the study primarily focused on the ISR pathway, potentially overlooking other mechanisms that contribute to the antitumor effects of syrosingopine and UK5099. Secondly, although our toxicity assessments have included H&E staining and liver and kidney function tests, further studies are needed to assess the dosing of dual pharmacological interventions.

In conclusion, our results demonstrated that the combination of syrosingopine and UK-5099 exhibited substantial antitumor activity in NSCLC. Furthermore, we unveiled the mechanism of the antitumor effect is through oxidative stress and metabolic dysfunction to sustainly activate the ISR. The combination of syrosingopine and UK-5099 may provide new insights into the treatment of NSCLC.

### Supplementary information

Supplementary materials

Original western blots

Supplementary Figure legends

## Data Availability

The data used to support the findings of this study are available from the corresponding author upon request.
