# Supplementary material for: Syrosingopine and UK5099 synergistically suppress non-small cell lung cancer by activating the integrated stress response
Source: Cell Death Dis. 2024 Jun 19;15(6):431. doi: 10.1038/s41419-024-06821-4 (PMC11187063; doi:10.1038/s41419-024-06821-4)
Supplement: Supplementary file 1 — Supplementary materials [file 41419_2024_6821_MOESM1_ESM.pdf]

Supplementary Table 1. Primer sequences for qRT-PCR.

Primer Name	Forward Primer Sequence (5' to 3')	Reverse Primer Sequence (5' to 3')
**MCT1**	TGGATGGAGAGGAAGCTTTCTAAT	CACACCAGATTTTCCAGCTTTC
**MCT4**	GCCATCTTTGCTGGTGGTTACC	TGGTCCAGAAAGGACAGCCATC
**MPC1**	CCACGCAACAAATGAAGTAGCCC	CTGGCAATGCTGTCCCTTCAAG
**MPC2**	CAATCTGCTGTTTTGATGGCTACA	CGAAAAAGCTGAGAGGCTCCTG
**ATF3**	CGCTGGAATCAGTCACTGTCAG	CTTGTTTCGGCACTTTGCAGCTG
**ATF4**	TTCTCCAGCGACAAGGCTAAGG	CTCCAACATCCAATCTGTCCCG
**BIP**	CATCACGCCGTCCTATGTCG	CGTCAAAGACCGTGTTCTCG
**CHOP**	TAAAGATGAGCGGGTGGCAG	GGATAATGGGGAGTGGCTGG
**GADD34**	TGAGACTCCCCTAAAGGCCA	CCAGACAGCCAGGAAATGGA
**TRIB3**	GCTTTGTCTTCGCTGACCGTGA	CTGAGTATCTCAGGTCCCACGT
**HRI**	ACCCCGAATATGACGAATCTGA	CAAGTGCTCCAGCAAAGAAAC
**PKR**	CCTGTCCTCTGGTTCTTTTGCT	GATGATTCAGAAGCGAGTGTGC
**PERK**	GCGGCAATGAGAAGTGGAAT	TCCCTCTGGGCTTAAAGGTG
**GCN2**	CAAGCTCAGCCAAGTCTACGTCATTC	GCTTCCACTTCTCTCCTAGTCAGCTTC
**GAPDH**	TGCACCACCAACTGCTTAGC	GGCATGGACTGTGGTCATGAG

Supplementary Table 2. siRNAs against HRI, PKR, PERK and GCN2.

Target Gene	siRNA1	siRNA2	siRNA3
**HRI**	GCACAAACTTCACG TTACT	AGAGCAATGTGGTG TTAAA	CCAGTTCAATTGTG GAACA
**PKR**	GGGATGGCATCATC TCAGA	GCGAGAAACTAGAC AAAGT	GGGATGGATTTGAT TATGA
**PERK**	GGAACGACCTGAAG CTATA	GGAAACAGCTATTC TCATA	GCATCTGCCTGGTT ACTTA
**GCN2**	GGACTAAAGTCACT GATGA	CCATCAACCTAACC CAGAA	GCAATTCTGTGGTG CATAA

Supplementary Table 3. Biochemical Values in Mice Serum

	Normal range	NC	Syrosingopine	UK-5099	Combination
AST（U/L）	36.31-235.48	134.8 ± 5.59	162.3 ± 22.85	168 ± 25.18	233.7 ± 12.73
ALT（U/L）	10.06-96.47	51.9 ± 0.3081	55.66 ± 2.481	57.09 ± 5.465	84.76 ± 23.55
BUN（mg/dL）	10.81-34.74	14.78 ± 0.9898	15.91 ± 16.11	16.11 ± 0.8351	18.91 ± 1.779
CREA（μmol/L）	10.91-85.09	17.59 ± 0.1813	18.23 ± 1.065	18.01 ± 1.279	20.84 ± 1.033

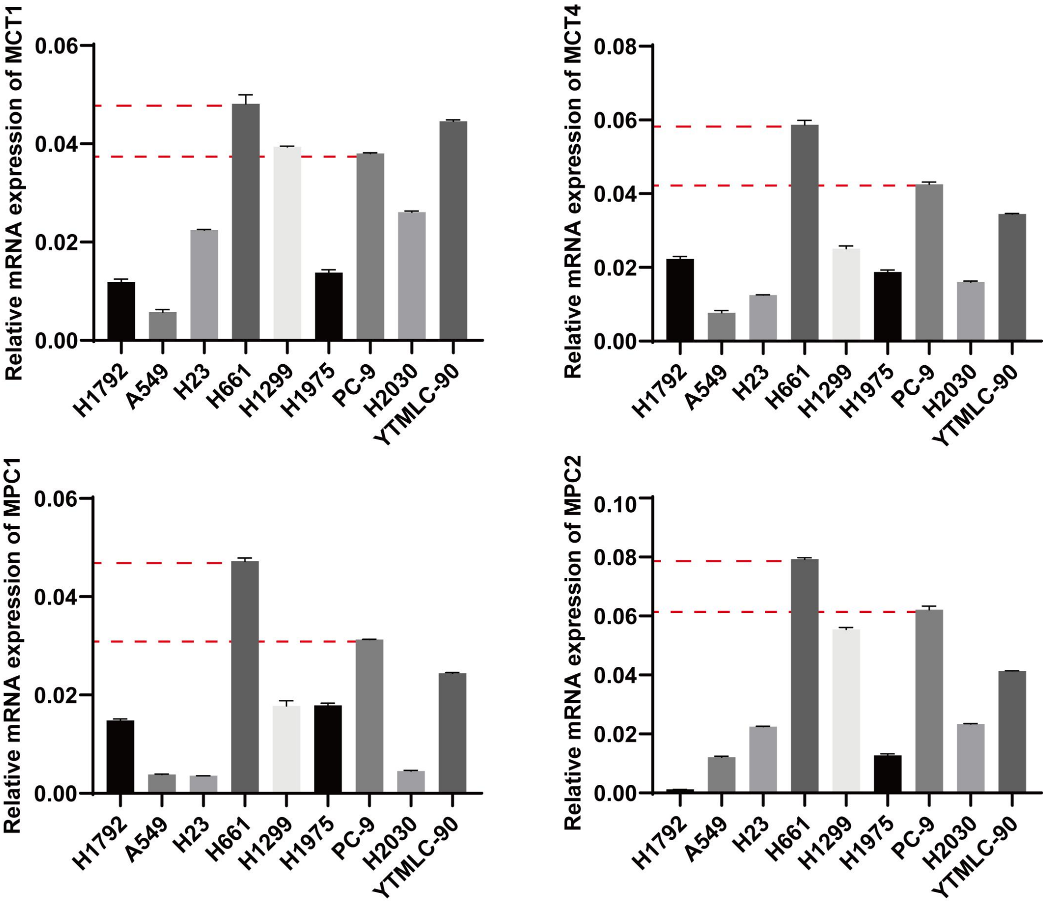

Figure S1. MCT1, MCT4, MPC1, and MPC2 mRNA expression levels in various human NSCLC cell lines (n=3).

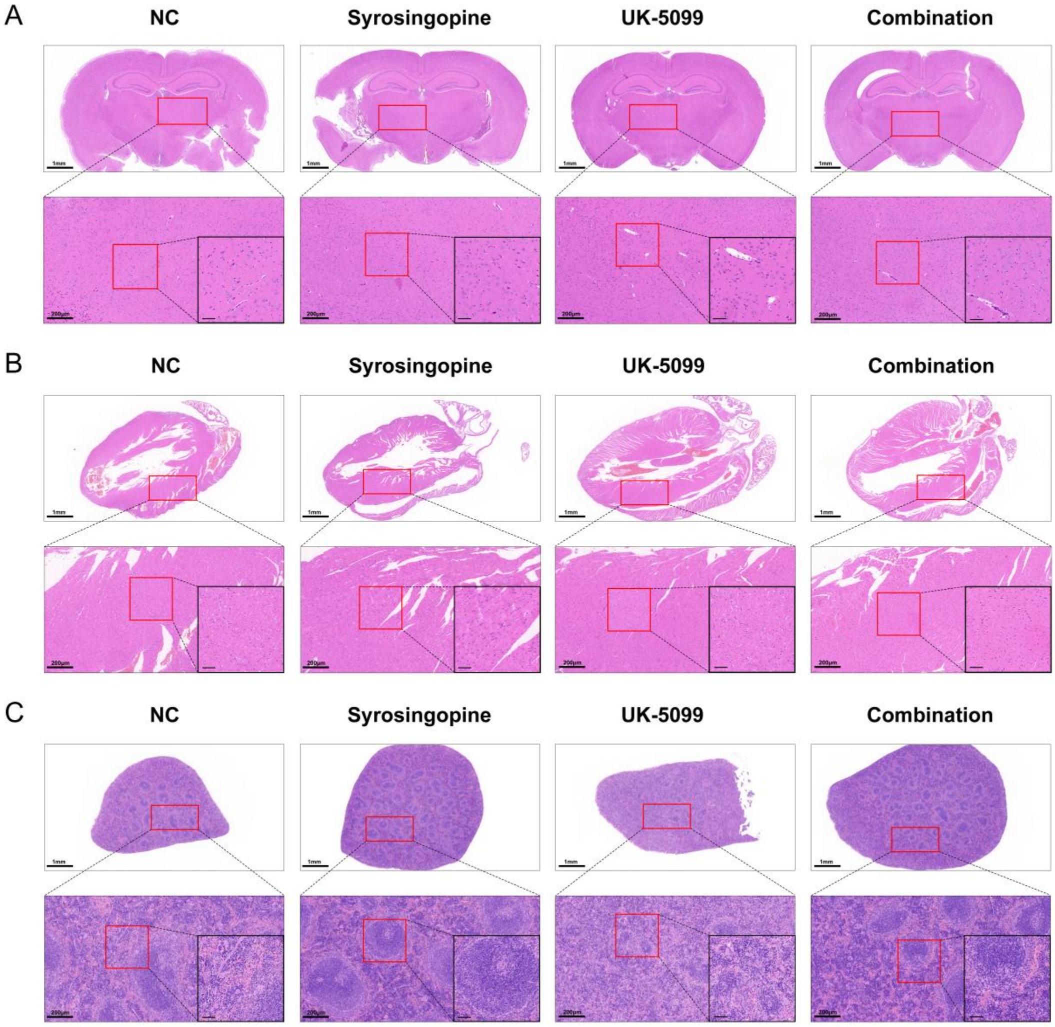

Figure S2. H&E staining of brain (A), heart (B) and spleen (C) tissues from mice at the therapeutic end point following different treatments (n=5).

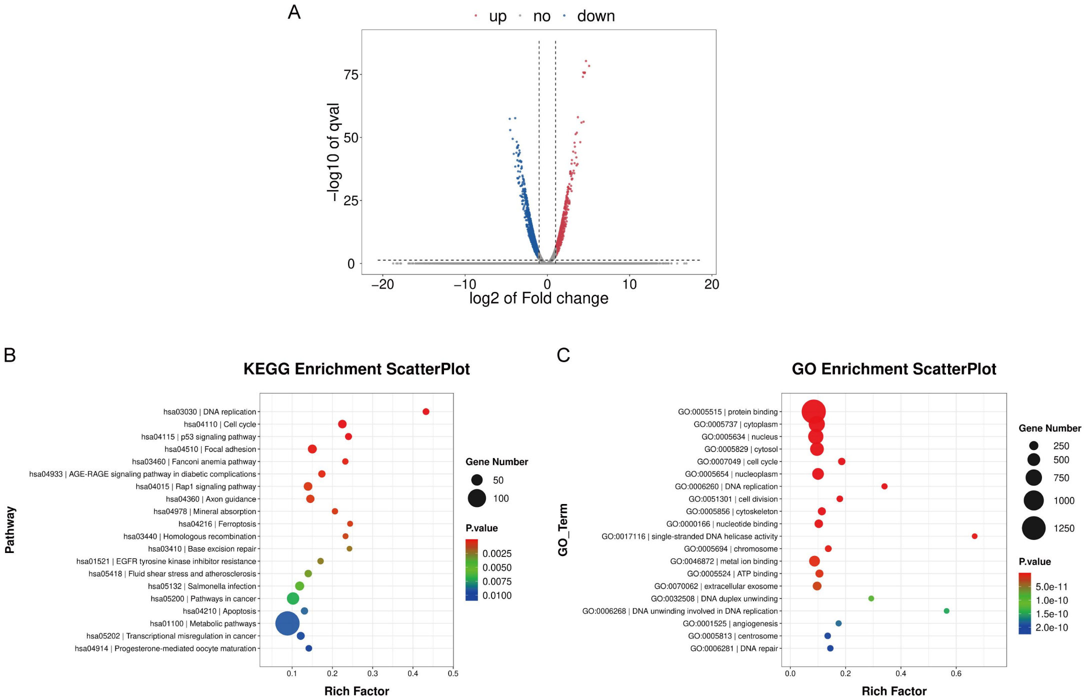

Figure S3. Comprehensive analysis of RNA-seq data depicting differential gene expression and enriched biological pathways (n=3). A. Volcano plot representing differentially expressed genes from RNA-seq data. B. KEGG analysis of differentially expressed genes from RNA-seq data. C. GO analysis of differentially expressed genes from RNA-seq data.

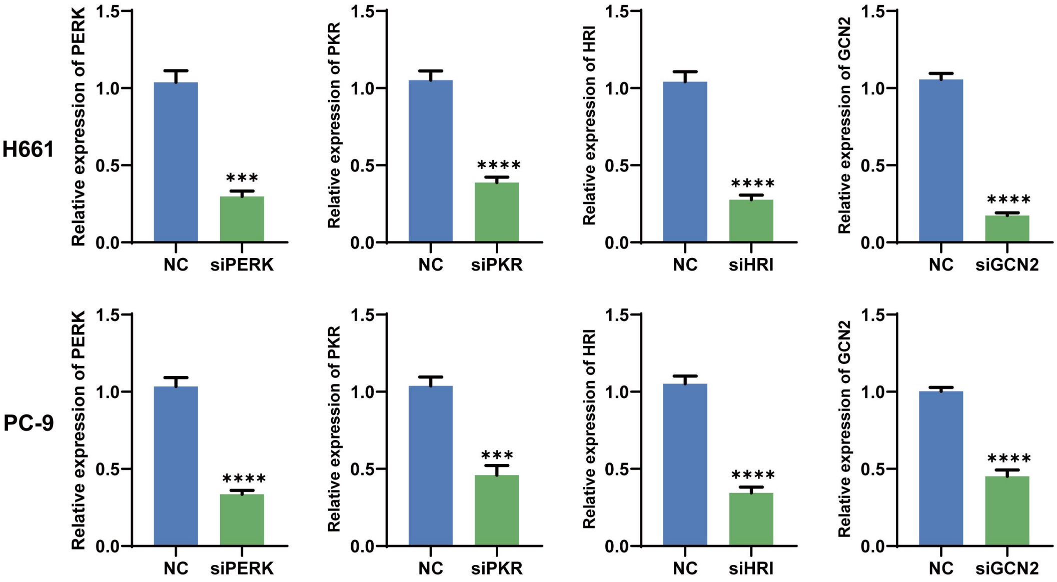

Figure S4. Knockdown of PERK, PKR, HRI, GCN2 was confirmed by quantitative RT-qPCR (n=3).
