# Supplementary figures and images for: Syrosingopine and UK5099 synergistically suppress non-small cell lung cancer by activating the integrated stress response
Source: Cell Death Dis. 2024 Jun 19;15(6):431. doi: 10.1038/s41419-024-06821-4 (PMC11187063; doi:10.1038/s41419-024-06821-4)

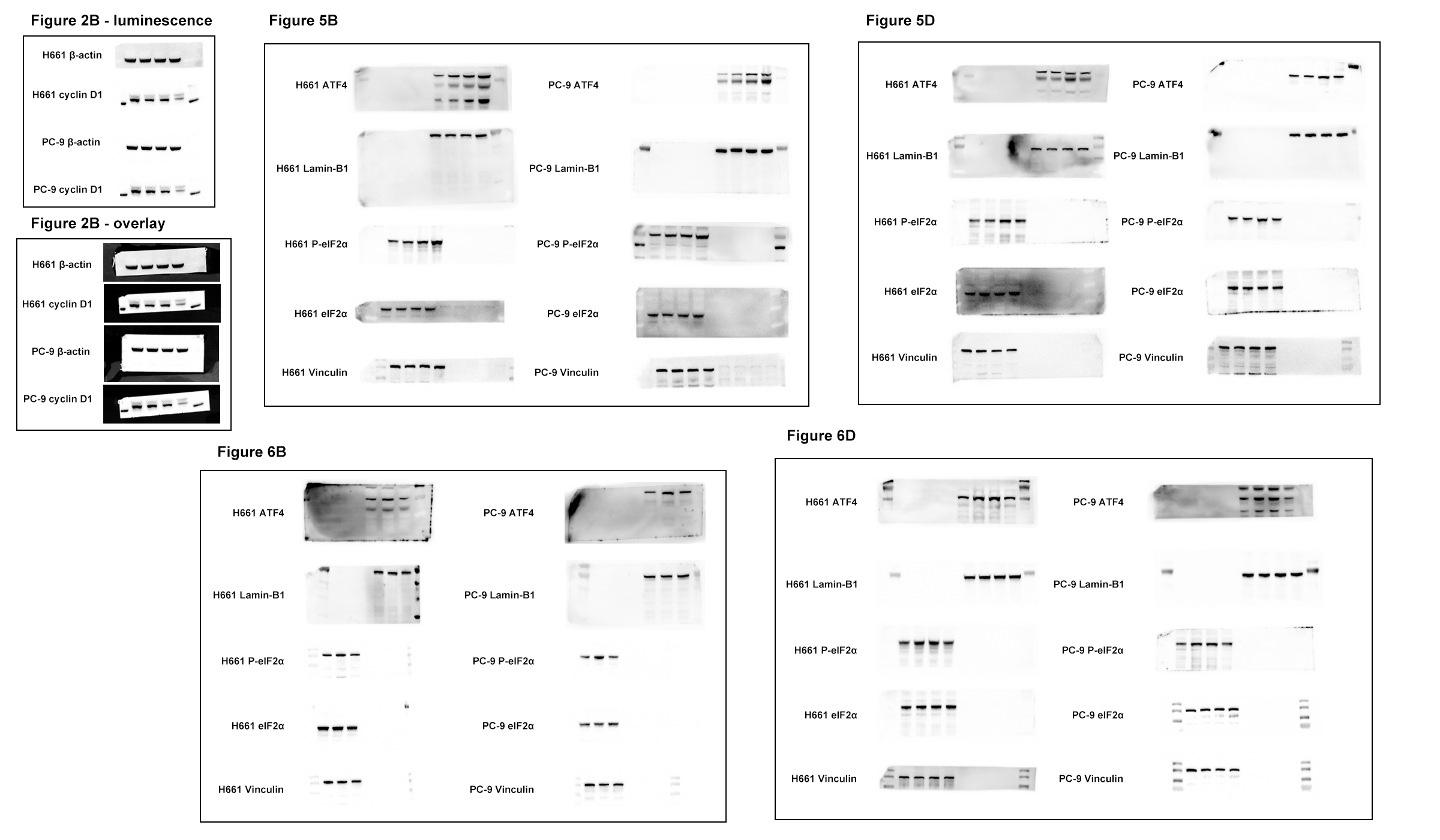

Supplement: Supplementary file 2 — Original western blots [file 41419_2024_6821_MOESM2_ESM.tif]
