# Supplementary material for: Syrosingopine and UK5099 synergistically suppress non-small cell lung cancer by activating the integrated stress response
Source: Cell Death Dis. 2024 Jun 19;15(6):431. doi: 10.1038/s41419-024-06821-4 (PMC11187063; doi:10.1038/s41419-024-06821-4)
Supplement: Supplementary file 3 — Supplementary Figure legends [file 41419_2024_6821_MOESM3_ESM.docx]

**Figure S1.** MCT1, MCT4, MPC1, and MPC2 mRNA expression levels in various human NSCLC cell lines (n=3).

**Figure S2.** H&E staining of brain (A), heart (B) and spleen (C) tissues from mice at the therapeutic end point following different treatments (n=5).

**Figure S3.** Comprehensive analysis of RNA-seq data depicting differential gene expression and enriched biological pathways (n=3). A. Volcano plot representing differentially expressed genes from RNA-seq data. B. KEGG analysis of differentially expressed genes from RNA-seq data. C. GO analysis of differentially expressed genes from RNA-seq data.

**Figure S4.** Knockdown of PERK, PKR, HRI, GCN2 was confirmed by quantitative RT-qPCR (n=3).

**Table S1.** Primer sequences for ~~q~~RT-PCR.

**Table S2.** siRNAs against HRI, PKR, PERK and GCN2.

**Table S3.** Biochemical Values in Mice Serum.
